# Serum Testosterone Levels and Androgen Receptor CAG Polymorphism Correlate with Hepatitis B Virus (HBV)-Related Acute Liver Failure in Male HBV Carriers

**DOI:** 10.1371/journal.pone.0084213

**Published:** 2013-12-31

**Authors:** Bao-Yan Xu, Wen-Ting Tan, Shun Tan, Yun-Jie Dan, Xiao-Li Luo, Guo-Hong Deng

**Affiliations:** 1 Department of Infectious Diseases, Southwest Hospital, Third Military Medical University, Chongqing, China; 2 Chongqing Key Laboratory for Research of Infectious Diseases, Chongqing, China; 3 Institute of Immunology, Third Military Medical University, Chongqing, China; George Mason University, United States of America

## Abstract

**Background:**

Augmentation of androgen/androgen receptor (AR) pathway may influence chronic hepatitis B (CHB) more likely in males. AR activity is modulated by a polymorphic CAG repeat sequence in AR exon 1. This study aimed to investigate the relationship between serum testosterone levels, CAG repeat numbers and hepatitis B virus (HBV)-related acute liver failure (ALF).

**Methods:**

Three hundred and seventy eight male CHB patients with ALF and 441 asymptomatic HBV carriers (AsCs) were recruited. AR CAG repeats numbers were analyzed. The serum testosterone levels of AsCs, ALFs and patients with hepatitis B flare groups, and sequential serum samples, were assessed quantitatively.

**Results:**

The median CAG repeat (M-CAG) frequency was significantly higher in ALF patients than AsCs (*P*<0.001). Patients with M-CAG alleles (*P*<0.001, OR 3.0, 95% CI 2.1–4.2) had the highest risk for ALF. Serum testosterone levels were significantly higher (*P*<0.001) at hepatitis flare point (8.2±3.0 ng/mL) than inactive phase (6.4±2.0 ng/mL). CHB (8.30±2.71 ng/mL, *P* = 7.6×10^−6^) and ALF group (2.61±1.83 ng/mL, *P* = 1.7×10^−17^) had significantly different levels of testosterone in comparison with AsCs group (6.56±2.36 ng/mL). The serum testosterone levels sharply decreased from hepatitis flare phase to liver failure phase, and tended to be normal at the recovery phase. Male AsCs with M-CAG alleles had significantly lower serum testosterone levels (*P*<0.05).

**Conclusions:**

There was a serum testosterone fluctuation during hepatitis B flare and HBV-related ALF, and the median CAG repeats in *AR* gene exon 1 were associated with lower serum testosterone levels in asymptomatic HBV carriers and an increased susceptibility to HBV-related ALF.

## Introduction

Hepatitis B virus (HBV) infection is a major health issue worldwide with estimated 350 million people chronically infected. It is the most common cause of liver cirrhosis and hepatocellular carcinoma in East and Southeast Asia [1]. Acute liver failure (ALF) is a unique presentation of chronic hepatitis B, characterized by very high alanine aminotransferase (ALT) levels accompanied by jaundice, and usually progresses to liver failure within 6 months [2], [3]. Spontaneous ALF is a life-threatening condition with a short-term mortality of 50–60% in China [4]. The pathogenesis of ALF is believed to be associated with vigorous immune response leading to excessive hepatic necroinflammation and decompensation. However, the underlying pathogenic mechanisms are currently unclear [5].

Male predominance is a remarkable clinical phenomenon in HBV-related liver diseases, including severe hepatitis B, liver cirrhosis and hepatocellular carcinoma (HCC) [6], [7]. Previous reports showed severe acute exacerbation developed predominantly in chronically infected men, with a male-to-female ratio of 4–12:1 [4], [8], [9]. Although alcohol intake may partially explain the gender difference, recent studies suggest that the androgen signal pathway plays a significant role in this male predominance. Earlier studies from Taiwan showed higher androgen levels and more active androgen receptor (AR) gene alleles correlated with an increased risk of HCC among male hepatitis B surface antigen (HBsAg) carriers [10], [11]. Serum testosterone levels markedly increase (20 to 30 folds higher) from puberty to young adulthood in males [12]. A cohort study showed earlier-onset puberty is associated with earlier HBeAg seroconversion, higher ALT levels, and a greater HBV viral load decrement in chronic HBV infected males [13]. Further independent studies elucidated the molecular mechanisms for the possible interactions between AR and HBV. HBV X protein (HBx) can enhance the transcriptional activity of AR in a ligand concentration-dependent manner through c-Src and glycogen synthase kinase-3beta kinase pathways [14]. Males were associated with higher HBsAg titer as well as intrahepatic replicative HBV DNA and transcripts in adult (10-week-old) but not prepubescent (4-week-old) mice [15]. AR can increase the transcription of HBV through direct binding to the cognate androgen-responsive element (ARE) sites in enhancer I (Enh I) of the HBV genome [16], and mutating the two androgen response elements within Enh I reduced HBV genome replication [15]. These findings imply that augmentation of the androgen/AR pathway may influence chronic hepatitis B more likely in males.

At the molecular level, the effect of androgens is mediated through the activation of AR. The activity of AR is modulated by a polyglutamine tract of variable size in its N-terminal transactivation domain. This polyglutamine tract is encoded by a highly polymorphic CAG repeat sequence in exon 1 of the *AR* gene located on chromosome X (Xq11.2) [17]–[19]. Recent studies showed that the length of the polymorphic CAG repeat in *AR* exon 1 is non-linearly correlated with AR transactivation activity in vitro, the ARs containing median CAG repeats displayed higher activity than ARs containing shorter and longer CAG repeats respectively [20], [21]. As the androgen/AR pathway seems to be important for the pathogenesis of hepatitis B, we propose that androgen/AR pathways may potentially impact the onset of HBV-related ALF. In this study, we investigated the androgen levels and the *AR* CAG repeat polymorphism in male chronic HBV carriers with HBV-related ALF and asymptomatic HBV carriers (AsCs).

## Materials and Methods

### Study participants

The flow diagram for patient recruitment in this study was depicted in Figure S1. We recruited 819 unrelated male Han Chinese HBV carriers from Southwest Hospital (Chongqing, China) between February 2001 and December 2010. All HBV carriers were positive for both HBsAg and IgG antibody to HBV core antigen (anti-HBc IgG) for at least 12 months. Liver function test, serum immunological markers screening and liver ultrasonography/computed tomography imaging were performed in all subjects. In the population, there is no serological evidence for coinfection or superinfection with hepatitis A, C, D, or E virus, cytomegalovirus (CMV), Epstein-Barr virus (EBV), and human immunodeficiency virus (HIV).

Among the 819 male HBV carriers, 441 asymptomatic carriers (AsCs, average age 41.3±12.7 years) and 378 patients with HBV-related ALF (ALFs, average age 40.5±10.2 years) were included. AsCs had normal serum levels of alanine aminotransferase (ALT), aspartate aminotransferase (AST), total bilirubin (TBil), peripheral blood leukocyte (4–10×10^9^/L) and platelet (100–300×10^9^/L) throughout the study, without any evidence of liver image/histological change and previous history of hepatitis B or liver cirrhosis. ALFs were defined according to the criteria: (1) An obvious hepatitis flare with serum ALT and TBil above 10 times the upper limit of normal (ULN); (2) Coagulation abnormality with international normalized ratio for prothrombin time test (INR) ≥1.5; (3) Without preexisting chronic liver diseases and with an illness of less than 26 weeks duration. Patients with liver cirrhosis were also included if both the ALF and liver cirrhosis had only been recognized for less than 26 weeks. (4) Negative for HAV, HEV, EBV and CMV IgM type antibodies, and negative for any HCV, HDV, and HIV antibodies. (5) Other causes including drug, herb, alcohol and ischaemia were also excluded.

### Ethics Statement

The study involved in the manuscript has been approved by the ethics committee of Southwest Hospital, Chongqing, China. All subjects provided written informed consent to participate in the study. The data were analyzed anonymously.

### CAG repeat polymorphism analysis

Genomic DNAs were extracted from peripheral blood leukocytes from 5 mL whole blood by using standard phenol/chloroform protocols. DNA samples were diluted to 8 ng/μL and distributed into 96-well plates (DNA panels), with 94 samples and 2 controls (DNA-free water) in each plate. The CAG trinucleotide repeats in exon 1 of the *AR* gene were analyzed as previously described [22]. Briefly, genomic DNAs were amplified with the forward primer 5′-CGGGGTAAGGGAAGTAGGTGGAAG-3′ (FAM labeled) and the reverse primer 5′-CTCTACGATGGGCTTGGGGAGAA C-3′. The amplification profile involved degeneration at 94°C for 2min; 30 cycles of 94°C for 30 s, 56°C for 30 s, 72°C for 40 s, then elongation at 72°C for 5 min. Polymerase chain reaction (PCR) products were loaded on a denaturing polyacrylamide gel and analyzed with an ABI 3730 genetic analyzer by GeneScan Analysis Software (Applied Biosystems, Foster City, CA). The fragment size was estimated by comparison with the internal size standard GS-LIZ500 (Applied Biosystems, Foster City, CA).

### Determination of serum total testosterone levels

Three groups of male patients were detected for serum testosterone levels, including 251 patients with AsCs, 32 patients with ALFs and 48 patients with mild to moderate hepatitis B (HBs). All patients with AsCs and ALFs were from above mentioned 819 male HBV carriers, who were genotyped with *AR* CAG repeats and had serum samples for tests. The 48 patients with mild to moderate hepatitis B were from our previous study [23], who had serum samples for tests. All other possible factors which may cause a hepatitis flare were excluded, including coinfection of other hepatitis related viruses, alcohol consumption, drug use. Screening from these patients with mild to moderate hepatitis B, we got 26 male patients with sequential serum samples both at inactive phase (with normal serum liver enzymes and bilirubin levels) and hepatitis B flare (ALT >5× ULN, TBil <5× ULN), the median time interval was 90 days (interval range from 36 to 630 days). One patient with sequential serum samples both at inactive phase (with normal serum liver enzymes and bilirubin levels) and severe hepatitis phase (TBil >10× ULN) was also included. Screening from patients with ALFs, we got three patients with sequential serum samples both at inactive phase (with normal serum liver enzymes and bilirubin levels) and ALF phase (TBil >10× ULN, coagulation abnormality with INR ≥1.5). The serum total testosterone levels of these samples were assessed quantitatively using commercial testosterone assay kit on Roche Elecsys 2010 chemiluminescence platform (Roche Diagnostics, Rotkreuz, Switzerland) according to manufacturer's protocol.

### Statistical analysis

Statistical analysis was performed using SPSS software (version 13.0; SPSS Inc, Chicago, IL). χ^2^ tests were performed to examine the differences in the CAG repeat number distribution between ALFs and AsCs groups. Logistic regression analysis was performed to adjust age and alcohol consumption. Kaplan-Meier survival analysis was used for evaluation the relationship between the age of ALF incidence and CAG repeats, and the Log Rank *P* value was calculated. The association between CAG repeats and the risk of ALF was estimated by *P* values, odds ratios (ORs), and 95% confidence interval (95% CI). Student *t* tests and one-way ANOVA were used to analyze the results of serum testosterone levels. A *P* value of less than 0.05 with two-tailed test was considered statistically significant.

## Results

### Case-Control Study

**Figure 1:**
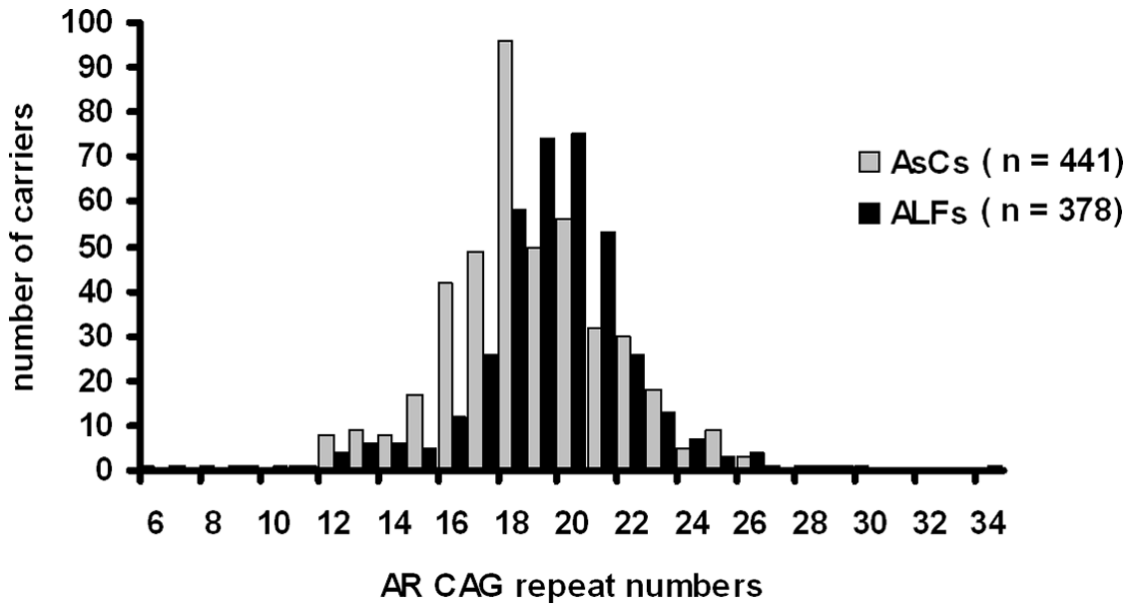
Distribution of AR CAG repeat alleles in male patients with HBV-related acute liver failure (ALFs) and asymptomatic HBV carriers (AsCs).

The clinical and demographic characteristics of the 819 unrelated male HBV carriers are summarized in Table 1. The ALF patients presented significant signs of hepatitis B flare (average peak ALT 842.0±821.2 IU/L) and liver failure (average peak TBil 477.7±185.9 μmol/L, average peak INR 2.60±1.26). There was no significant difference between age (*P* = 0.289) and alcohol use (*P* = 0.969) between the AsCs and the ALF patients. The percentage of HBeAg positive was lower in the AsCs group than in the ALF group (22.0% vs. 31.0%, *P*<0.01). For the 819 men, the *AR* CAG repeats ranged from 6–34 presenting a normal distribution and a peak at 18–20 (Figure 1). The mean CAG repeat numbers was 19.0±2.9 for all male participants, there was a significant difference for mean CAG repeat numbers between ALF patients and AsCs (Table 1, 19.4±2.7 vs. 18.6±3.0, *P*<0.001).

**Table 1:** Clinical characteristics of the 819 unrelated male HBV carriers enrolled in the study.

	ALFs			AsCs (n = 441)	*P* values §
	LC-ALFs (n = 179)	NLC-ALFs (n = 199)	All ALFs (n = 378)		
Age (years, mean ± SD)	43.1±12.9	39.7±12.3	41.3±12.7	40.5±10.2	0.289
ALT (IU/L, mean ± SD)	747.5±819.6	921.8±816.4	842.0±821.2	30.0±11.7	
TBil (μmol/L, mean ± SD)	479.9±194.3	476.0±179.2	477.7±185.9	14.5±5.0	
INR (mean ± SD)	2.19±0.82	2.39±0.93	2.29±0.88	-	
HBeAg positive, n (%)	52 (29.1)	65 (32.7)	117 (31.0)	97 (22.0)	0.0047
CAG repeat numbers (mean ± SD)	19.4±2.4	19.4±3.0	19.4±2.7	18.6±3.0	0.0002
Alcohol drink, n (%)					0.969
Never	112 (62.6)	113 (56.8)	225 (59.5)	262 (59.4)	
Occasionally	26 (14.5)	53 (26.6)	79 (20.9)	102 (23.1)	
Mild (<50g/d)	16 (8.9)	12 (6.0)	28 (7.4)	26 (5.9)	
Heavy (≥50g/d and <100g/d)	10 (5.6)	7 (3.5)	17 (4.5)	16 (3.6)	
Problem (≥100g/d)	15 (8.4)	14 (7.0)	29 (7.7)	35 (7.9)	

ALFs, HBV-related acute liver failure. AsCs, asymptomatic HBV carriers. LC-ALFs, ALF patients with liver cirrhosis. NLC-ALFs, ALF patients without liver cirrhosis. HBV, hepatitis B virus. HBeAg, hepatitis B virus e antigen. ALT, alanine aminotransferase. TBil, total bilirubin. INR, international normalized ratio for prothrombin time test. SD, standard deviation. §, comparison is conducted between AsCs and ALFs groups, with χ^2^ tests for HBeAg positive and alcohol drink rates, and student's t-tests for age, HBV DNA and CAG repeat numbers.

**Figure 2:**
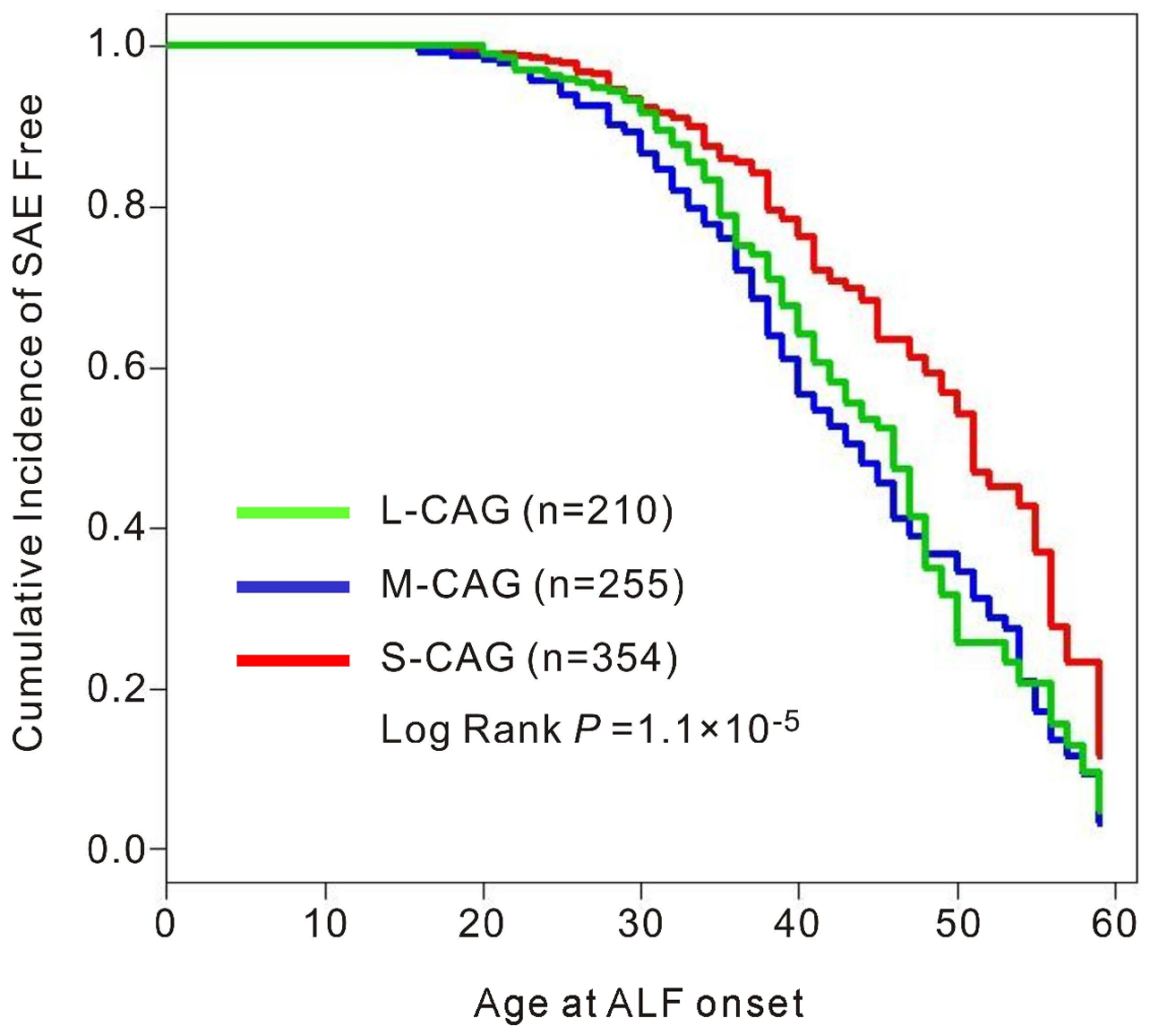
Relationship between the onset age of HBV-related acute liver failure (ALFs) and *AR* CAG repeat categories. Kaplan–Meier survival curves demonstrating age of ALF occurrence based on *AR* CAG repeat alleles: S-CAG (CAG repeat number <19, red line), M-CAG (CAG repeat number 19–20, blue line) and L-CAG (CAG repeat number >20, green line). *P* value based on Log Rank test is given.

We then divided the CAG allele lengths into three categories for the stratified analysis: short (<19, S-CAG), median (19–20, M-CAG) and long (>20, L-CAG). The CAG repeat allele distributions and frequencies in ALF patients and the AsCs group were summarized in Table 2. The M-CAG allele frequencies were significantly higher in ALF patients than those in AsCs (39.4% vs. 24.0%, *P*<0.001). The association remained significant in both ALF groups with and without liver cirrhosis (for LC-ALFs, *P*<0.001; for NLC-ALFs, *P*<0.001). Logistic regression analysis with adjustment for age, alcohol consumption and HBeAg status indicated that *AR* CAG polymorphism was independently associated with ALF. Patients with M-CAG alleles (*P*<0.001, odds ratio (OR) 3.0, 95% CI 2.1–4.2) or L-CAG alleles (*P*<0.001, OR 2.3, 95% CI 1.6–3.3) had a significantly increased risk for ALF. We further analyzed the role of *AR* CAG repeats on the occurrence age of ALF with the Kaplan–Meier survival analysis (Figure 2). Significant differences were found between L-CAG, M-CAG and S-CAG allele in total case-control cohort (Log Rank test *P*<0.001), in which the patients with M-CAG alleles had a highest risk for ALF.

**Table 2:** Distributions of patients with HBV-related acute liver failure (ALFs) and asymptomatic HBV carriers (AsCs) according to the qualitative categories of *AR* CAG repeats.

	S-CAG allele (repeat numbers <19)	M-CAG allele (repeat numbers 19–20)	L-CAG allele (repeat numbers >20)	*p* values
AsCs, n (%)	234 (53.1)	106 (24.0)	101 (22.9)	
ALFs, n (%)	120 (31.8)	149 (39.4)	109 (28.8)	2.4×10^−9^
LC-ALFs, n (%)*	49 (27.4)	80 (44.7)	50 (27.9)	6.1×10^−9^
NLC-ALFs, n (%)*	71 (35.7)	69 (34.7)	59 (29.6)	2.2×10^−4^
OR (95% CI)	1.0 (reference)	3.0 (2.1–4.2)	2.3 (1.6–3.3)	1.3×10^−9^

LC-ALFs, ALF patients with liver cirrhosis. NLC-ALFs, ALF patients without liver cirrhosis.

### Serum testosterone levels in patients with hepatitis B

**Figure 3:**
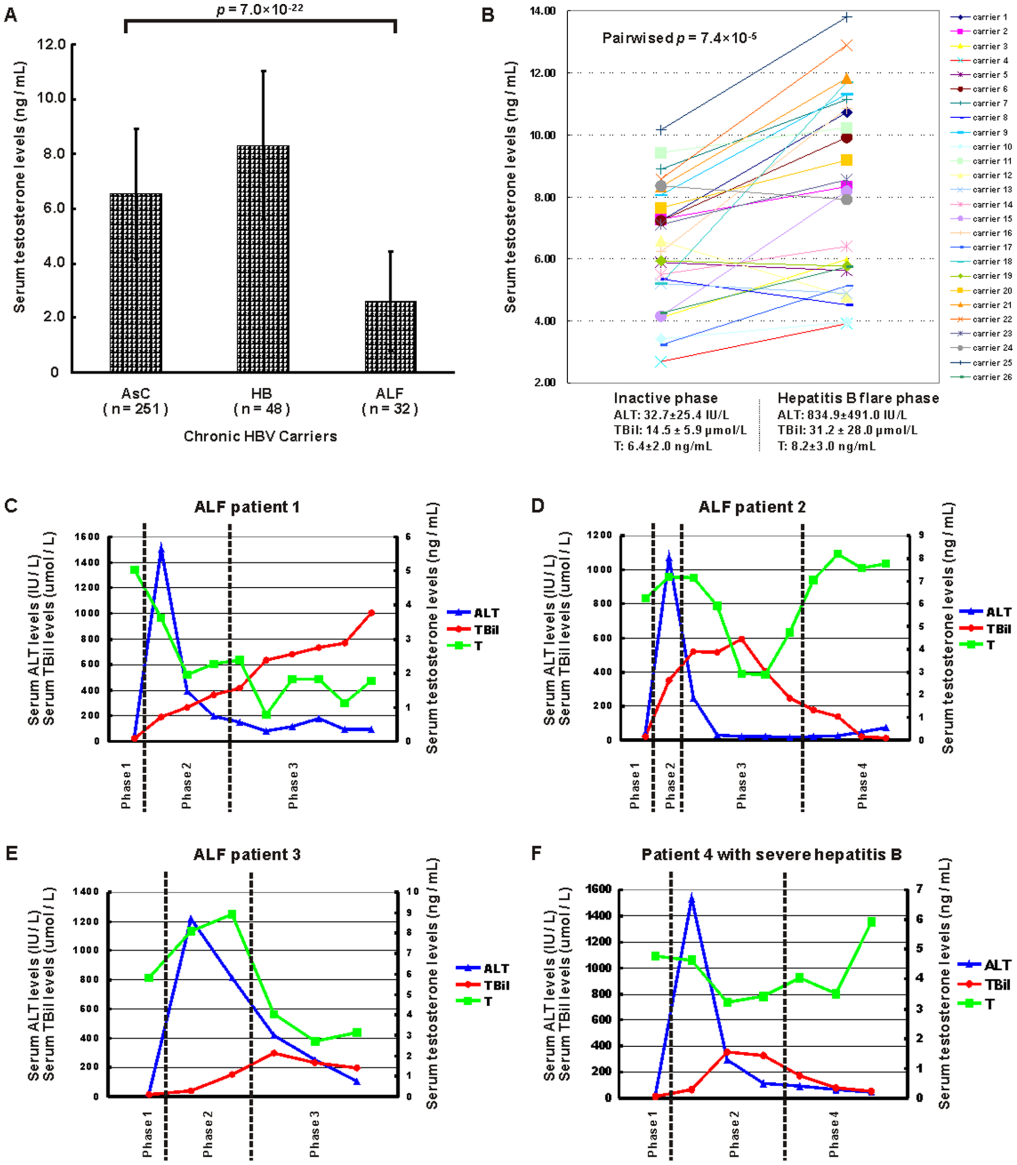
Fluctuations of serum testosterone levels at different phases of chronic hepatitis B. (A) The serum testosterone levels in three male HBV carriers groups. AsC, asymptomatic HBV carriers. HB, patients with mild to moderate hepatitis B flare. ALF, patients with HBV-related acute liver failure. *P* value based on one-way ANOVA was given. (B) Serum testosterone levels in 26 male chronic hepatitis B patients with sequential serum samples both at inactive phase and hepatitis B flare. ALT, alanine aminotransferase. TBil, total bilirubin. T, testosterone. (C–F) Serum testosterone levels in four patients with sequential serum samples both at inactive phase (with normal serum liver enzymes and bilirubin levels) and severe hepatitis phase (TBil >10× ULN). ALT, alanine aminotransferase. TBil, total bilirubin. T, testosterone. ALF, patients with HBV-related acute liver failure. Phase 1, inactive phase. Phase 2, hepatitis flare phase. Phase 3, liver failure phase (TBil >10× ULN, coagulation abnormality with INR ≥1.5). Phase 4, recovery phase.

We detected the serum testosterone levels in three male HBV carriers groups, including 251 male AsCs (average age 43.1±7.6 years, average ALT 29.6±10.5 IU/L, average TBil 14.9±10.3 μmol/L), 48 patients with mild to moderate hepatitis B (average age 34.4±9.5 years, average ALT 659±509 IU/L, average TBil 25.0±15.4 μmol/L), and 32 patients with ALFs (average age 42.2±12.8 years, average ALT 362±538 IU/L, average TBil 358.7±229.2 μmol/L, average INR 2.39±1.32). Patients with mild/moderate hepatitis B flare (HB group) had a significantly higher levels of testosterone (8.30±2.71 vs. 6.56±2.36 ng/mL, *P* = 7.6×10^−6^) in comparison with AsCs group (Figure 3A). However, ALF group had a significantly lower levels of testosterone (2.61±1.83 vs. 6.56±2.36 ng/mL, *P* = 1.7×10^−17^), which indicated an elevation of serum testosterone levels during hepatitis B flare and a decrease of serum testosterone levels during acute liver failure (Figure 3A). To confirm this, we further observed this trend in sequential serum samples from baseline to hepatitis B duration in each patient. We detected the serum testosterone levels in 26 male chronic hepatitis B patients (average age 30.7±6.7 years) with sequential serum samples both at inactive phase (average ALT 42.7±25.4 IU/L, average TBil 14.5±5.9 μmol/L) and hepatitis B flare (average ALT 834.9±491.0 IU/L, average TBil 31.2±28.0 μmol/L). We observed a testosterone pulse trend between inactive phase and hepatitis flare phase (Figure 3B). The serum testosterone levels were significantly higher at hepatitis flare point (mean 8.2±3.0 ng/mL) than those at inactive phase (mean 6.4±2.0 ng/mL) for pairwise comparison (*P*<0.001). For the three patients with sequential serum samples both at inactive phase (with normal serum liver enzymes and bilirubin levels) and ALF phase (TBil >10× ULN, coagulation abnormality with INR ≥1.5), the serum testosterone levels sharply decreased from hepatitis flare phase to liver failure phase (Figure 3C–E), and elevated from liver failure phase to recovery phase (Figure 3D). The variation pattern of serum testosterone levels was similar in a patient with severe hepatitis B (Figure 3F).

**Figure 4:**
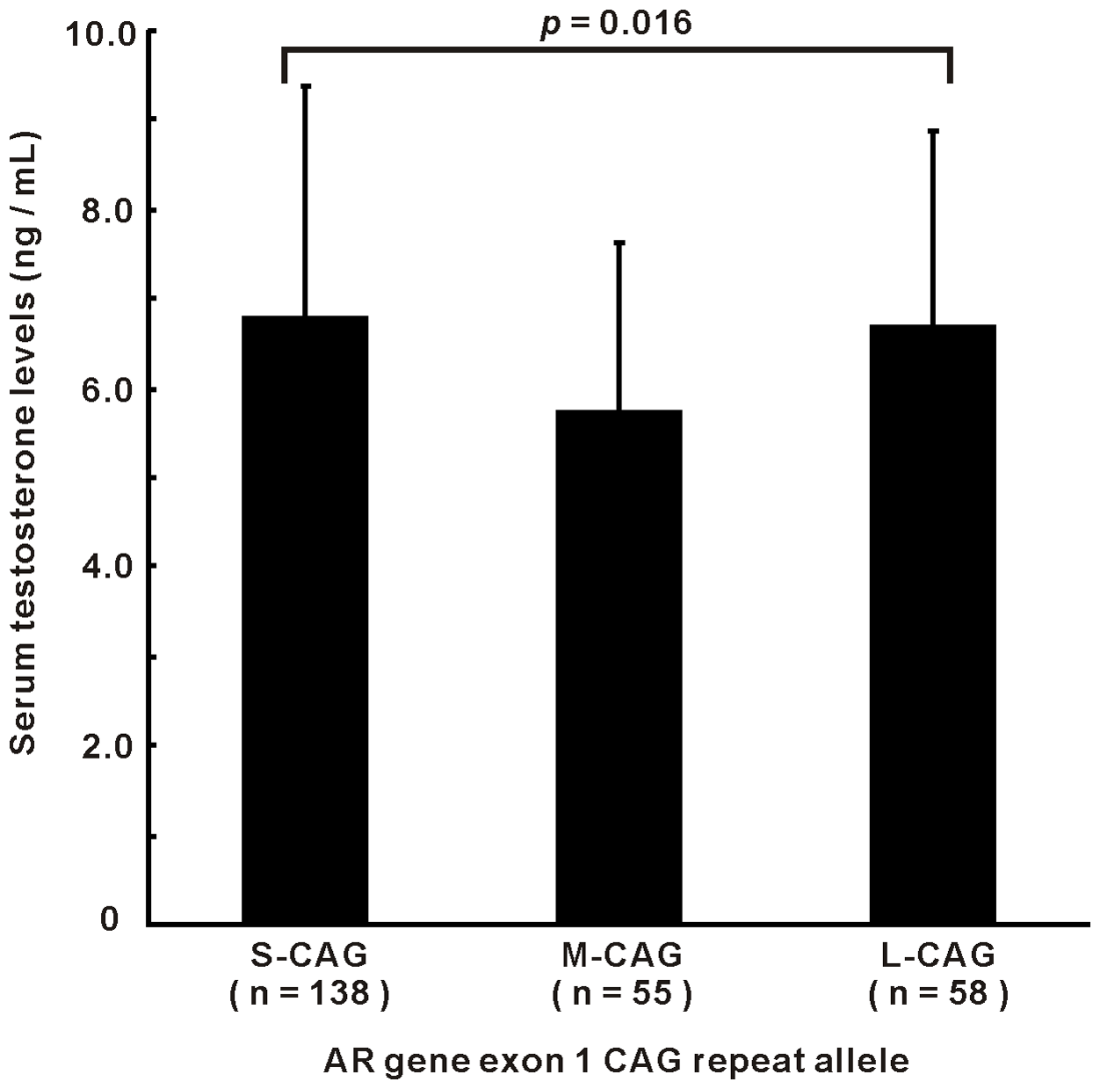
Correlation between serum testosterone levels and AR CAG repeat length. A total of 251 male asymptomatic HBV carriers (AsCs) with normal liver enzyme levels were detected for serum testosterone levels. *AR* gene alleles were categorized by exon 1 CAG repeat numbers: S-CAG (CAG repeat number <19), M-CAG (CAG repeat number 19–20) and L-CAG (CAG repeat number >20). *P* value based on one-way ANOVA was given.

We also evaluated the relationship between the *AR* CAG repeat and serum testosterone levels in the above 251 male AsCs. We found that there was a non-linear association between *AR* CAG repeat and serum testosterone levels. Men with M-CAG alleles had significantly lower serum testosterone levels (Figure 4, *P* = 0.016).

## Discussion

We had screened the *AR* genomic region by PCR-resequencing in 32 Chinese and found no common single nucleotide polymorphisms in exons or promoter region of *AR* gene (unpublished data). CAG repeat on exon 1of *AR* gene, which encodes a polymorphic uninterrupted polyglutamine (poly Q) tract, is the major polymorphic locus of *AR* gene. It has been extensively studied for association in a variety of male predominant diseases [11], [21], [24], such as male-type alopecia, male infertility, prostate cancer, HBV-related hepatocellular carcinoma. We present here a clinical and laboratory dataset of *AR* CAG repeat and serum testosterone level measurements in male chronic hepatitis B patients.

We demonstrated that there was a serum testosterone fluctuation during hepatitis B flare and HBV-related ALF, and the median CAG repeats in *AR* gene exon 1 were associated with lower serum testosterone levels in asymptomatic HBV carriers and an increased susceptibility to HBV-related ALF. Our results indicate a significant correlation between androgen pathway and HBV-related ALF for the first time.

Most previous association studies between *AR* CAG length and male predominance diseases were analyzed with a linear model because of a general opinion that the CAG tract is inversely associated with AR activity. However, recent studies have shown that the AR activity measured in vitro is much dependent on the experimental setting. Following adjustment for AR protein, the median number of CAG tract (M-CAG) confine optimal receptor function compared with both shorter and longer repeats [20]. Our results showed the M-CAG allele had the highest risk (about 3-fold to S-CAG alleles and 1.3-fold to L-CAG alleles) for ALF, which is concordant with the non-linear association between *AR* CAG repeat length and risk of subfertility [21] or polycystic ovary syndrome [25]. Interestingly, we also observed asymptomatic HBV carriers with the M-CAG alleles had lower serum testosterone levels, which indicated decrease in AR activity may be compensated by parallel changes in the level of testosterone. This is in agreement with the cross-sectional results in 2878 European men from European Male Ageing Study (EMAS). The longer *AR* CAG repeat length correlates significantly with higher total, free, and bioavailable levels of testosterone in men [26]. The *AR* CAG stretch is in the protein's transactivating domain (N-terminal), which interacts with the hormone-binding domain (C-terminal). The interplay between the transactivating and the hormone-binding domains had previously been shown to be significantly reduced by shorter or longer CAG repeats than by the normal range [27]. This mechanism implied both increased and decreased CAG length might attenuate AR function.

We observed the serum testosterone levels increased during chronic hepatitis B flare, decreased with the severity of hepatitis B (especially at liver failure phase), and tended to be normal at the recovery phase. The fluctuation pattern was robust both in phenotypic groups and sequential serum samples from inactive baselines to different hepatitis phases (flare, liver failure and recovery stages). The functional relevance between serum testosterone pulse and elevated levels of liver enzymes remain unclear in this study. There is likely an association between testosterone levels and the host immune response [28], [29], which is responsible for the activation of immune injury to the liver. Moreover, AR can increase the transcription of HBV through direct binding to the cognate androgen-responsive element sites in enhancer I of the HBV genome [15], [16]. Thus, a plausible explanation could be that the testosterone pulse may promote AR-specific HBV replication and immune activation (both factors contribute to the pathogenesis of acute exacerbation of liver necroinflammation), and HBV carriers with median CAG repeats are more sensitive to the testosterone pulse because of the highest AR transcription activity and sharp elevation from a relatively lower baseline testosterone levels compared with carriers with shorter or longer CAG repeats. Our study emphasizes the importance of androgen/AR action in the pathogenesis of severe hepatitis B.

In conclusion, our clinical and laboratory investigation suggested the androgen/AR could be a part of the host variation which underlies the phenotypic variation seen in individuals' susceptibility to HBV-related ALF in the Chinese population. However, further studies are required to ascertain the importance of the androgen/AR actions in HBV-related ALF. First, our conclusion needs support by data from other populations. Second, it is also interesting to investigate whether the *AR* CAG repeats and androgen levels are also associated with HBV-related ALF in women. Moreover, the molecular mechanisms of androgen/AR action on the pathogenesis of HBV-related ALF need to be further identified. With more uncovered biological mechanisms of androgen/AR action involved, we may translate the androgen/AR action into clinical practice (surveillance, intervention, therapy) for patients with HBV-related ALF rather than an aspirational goal.

## Supporting Information

Figure S1 **The flow diagram for patient recruitment in this study.** Blue boxes indicates the patients genotyped for AR CAG repeat, and red boxes indicates the patients tested for serum testosterone levels. AsC, asymptomatic HBV carriers. HB, patients with hepatitis B flare. ALF, patients with HBV-related acute liver failure. HAV, hepatitis A virus. HCV, hepatitis C virus. HDV, hepatitis D virus. HEV, hepatitis E virus. HCMV, human cytomegalovirus. EBV, Epstein-Barr virus. HIV, human immunodeficiency virus. DILI, drug induced liver injury. AIH, autoimmune hepatitis.
(TIF)
