# Supplementary figures and images for: Serum Testosterone Levels and Androgen Receptor CAG Polymorphism Correlate with Hepatitis B Virus (HBV)-Related Acute Liver Failure in Male HBV Carriers
Source: PLoS One. 2013 Dec 31;8(12):e84213. doi: 10.1371/journal.pone.0084213 (PMC3877261; doi:10.1371/journal.pone.0084213)

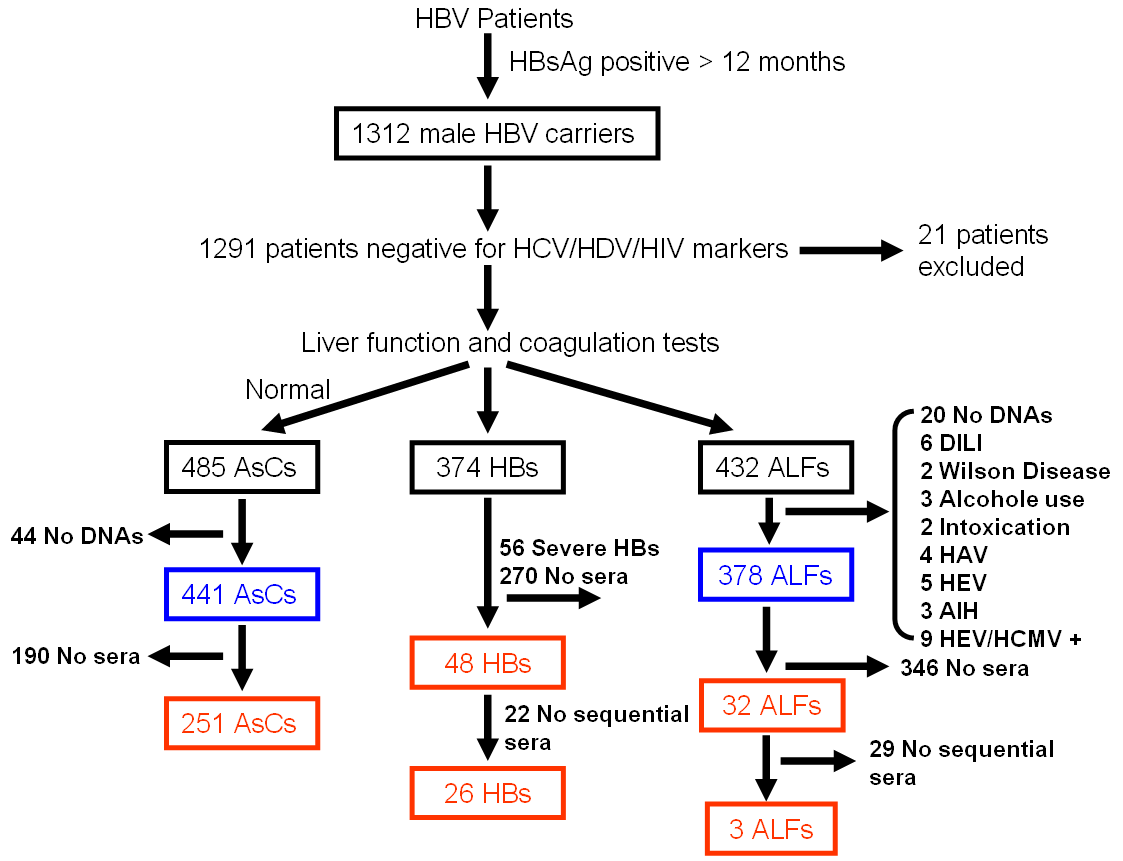

Supplement: Figure S1 — The flow diagram for patient recruitment in this study. Blue boxes indicates the patients genotyped for AR CAG repeat, and red boxes indicates the patients tested for serum testosterone levels. AsC, asymptomatic HBV carriers. HB, patients with hepatitis B flare. ALF, patients with HBV-related acute liver failure. HAV, hepatitis A virus. HCV, hepatitis C virus. HDV, hepatitis D virus. HEV, hepatitis E virus. HCMV, human cytomegalovirus. EBV, Epstein-Barr virus. HIV, human immunodeficiency virus. DILI, drug induced liver injury. AIH, autoimmune hepatitis. (TIF) [file pone.0084213.s001.tif]
